# Endogenous extraction yielded high quality sulforaphane from broccoli sprouts unveils potent antioxidant and anti-Alzheimer's activities

**DOI:** 10.1016/j.heliyon.2025.e42673

**Published:** 2025-02-13

**Authors:** Shyam Kokkattunivarthil Uthaman, Wan Seok Kang, Ju-Young Park, Sunoh Kim, Duc Dat Le, Suk-Jung Oh, Karthik Murugesh, Laura Minju Oh, Mina Lee, Jin Woo Park

**Affiliations:** a Research & Development, Ecoworld Pharm Co. Ltd., Damyang, 57304, Republic of Korea; b Central R&D Center, B&Tech Co., Ltd., Naju, 58025, Republic of Korea; c College of Pharmacy and Research Institute of Life and Pharmaceutical Sciences, Sunchon National University, Suncheon, 57922, Republic of Korea; d Department of Natural Cosmetics Science, Graduate School, Sunchon National University, Suncheon, 57922, Republic of Korea; e Department of Biomedicine, Health & Life Convergence Sciences, BK21 Four, Biomedical and Healthcare Research Institute, Mokpo National University, Jeonnam, 58554, Republic of Korea

**Keywords:** Sulforaphane, Broccoli sprout extract, Alzheimer's disease, Cruciferous vegetables, Antioxidant activity, Endogenous extraction

## Abstract

The extraction of sulforaphane (SFN) is challenging due to its instability and low water solubility, with existing methods often involving toxic solvents or yielding low SFN. We optimized an endogenous extraction protocol for high SFN content, characterized by HPLC and LC-MS analyses. SFN remained stable in refrigerated broccoli sprout extract powder (BSEP) for over a month. BSEP showed four times higher oxygen radical absorbance capacity (ORAC) than the SFN standard, indicating high antioxidant capacity. It also reduced inflammatory responses by down-regulating COX-2, IL-6, and TNF-α gene expression in LPS-induced RAW 264.7 macrophages. Additionally, BSEP exhibited neuroprotective properties in amyloid-beta (1−42) (Aβ_1−42_)-induced Alzheimer's disease (AD) mice, enhancing memory and learning retention in water maze and passive avoidance tests. BSEP mitigated spatial cognitive impairment and improved memory function in Aβ_1−42_-induced memory-deficient mice. While BSEP did not alter acetylcholine (ACh) concentration, it improved memory and learning by inhibiting acetylcholinesterase (AChE) activity. BSEP with SFN content exceeding 200 mg/kg ameliorated neurobehavioral deficits and protected the brain from amyloid deposition, suggesting its therapeutic potential in AD treatment. We propose an eco-friendly form of SFN-rich BSEP for daily intake and commercial therapeutics.

## Graphical abstract

**Figure:**
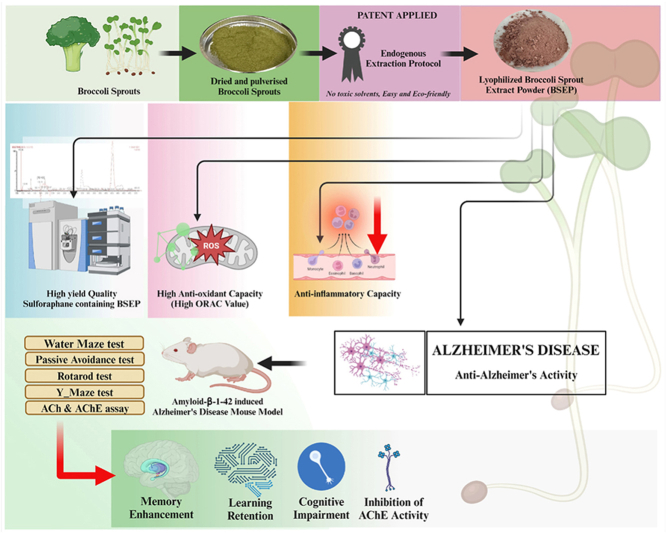

## 1 Introduction

Sulforaphane (1-isothiocyanato-4-methylsulfonylbutane, SFN), a well-researched dietary bioactive isothiocyanate compound, serves as a metabolite of glucoraphanin, a phytonutrient abundantly present in cruciferous vegetables like broccoli, Brussels sprouts, and cabbage exhibits antioxidant, anti-inflammatory, and detoxification properties [[1], [2], [3]]. Chemically, the SFN contains an isothiocynate functional group (-N=C=S) and a methylsufonyl side chain (R-(S-O)-R) [4] and which is formed naturally by the hydrolytic action of an endogenous thioglucosidase enzyme known as myrosinase acted on stored precursor glutathione upon physical damage to the cell [4]. Remarkable water solubility owing to its electrophilic structure and absence of aromatic groups enhancing its pharmacological activity within the neutral pH environment of the intestine [5]. Extensive research indicates that a significant intake of cruciferous vegetables is linked to a decreased risk of total mortality, cardiovascular diseases and highly neuroprotective [3,[6], [7], [8]]. It modulates the nuclear factor-kappa B (NFkB) pathway, playing a crucial role in DNA transcription and pro-inflammatory cytokine production, and is produced upon the interaction of myrosinase with glucoraphanin [4]. Also, it exhibits potent activation of the cellular antioxidant pathway Keep1/Nrf2/ARE and other anti-inflammatory mechanisms by inhibiting the Nfkb pathway [9,10]. The hormetic activation of Nrf2 by SFN presents an opportunity to potentially mitigate a diverse array of neurological pathologies in experimental models of human diseases such as Alzheimer's disease (AD) [11], Parkinson's disease [12], Huntington's disease [13], amyotrophic lateral sclerosis [14], multiple sclerosis [15], autism spectrum disorder (ASD) [16], and schizophrenia [17]. Additionally, it demonstrates anti-inflammatory properties by binding to toll-like receptor-4 (TLR4) and blocking the downstream transcription factor NFkB, which regulates pro-inflammatory cytokines [18,19].

In animal studies, SFN has often been administered through intraperitoneal injections, despite the typical oral route of administration [2]. However, daily dietary intake of SFN can offer the same noted benefits. For human consumption, the recommended daily dosage of SFN is 2–6 mg/day for adults (2), as doses exceeding 150 mg/kg body weight may lead to toxicity [2]. While cruciferous vegetables contain SFN, the quantity required to meet these recommended doses is impractical for a regular diet [20]. To address this challenge, there is a need for a naturally derived concentrated form of SFN that can be taken orally as a nutrient supplement. Extracting naturally occurring SFN from its source is difficult due to its high instability [21]. Bioactive compound extraction from cruciferous vegetables is a significant challenge and the information regarding the extraction process from Broccoli is scarce [22]. These methods include conventional solvent extraction preferably aqueous and non-conventional methods such as high-pressure processing (HPP), high voltage electrical discharges (HVED), ultrasound-assisted extraction (UAE), microwave-assisted extraction (MAE), supercritical fluid extraction (SFE), and pressurized fluid extraction (PFE) [23], with regular steps like blanching, water bath incubation and freeze-drying [[24], [25], [26]]. Although efforts towards the application of eco-friendly non-conventional extraction methods have been made, the use of conventional solvent extraction is still prevailing due to the quality and moreover stable yield of SFN [23]. All of these extraction uses risky, toxic and expensive chemicals such as methanol, methylene chloride, ethyl acetate which are not safe in foods [24,27]. Furthermore, the unstable nature of SFN has prompted the development of stabilized preparations, including an a-cyclodextrin-encapsulated form of SFN and a stabilized version of pure plant-derived SFN known as Prostaphane® [2].

Potent antioxidant and anti-inflammatory capacity of the SFN promises AD treatment by improving brain health. SFN prevents Aβ production, reducing levels of *Tau* protein, inhibits the levels of inflammatory, oxidative stress, synaptic damage and neurodegeneration biomarkers and ameliorating cognatic impairment in the animals [3]. This multifaceted neuroprotective effect via antioxidant and anti-inflammatory ability must be possessed by the SFN extracted and stabilized. Indeed, extracted SFN should be evaluated for these characteristic capacities to be used in clinical applications such AD management [3,28,29]. In nutshell, a simple and economically viable extraction method from available vegetable sources is required. It is crucial to ensure that the extraction protocol renders SFN stable for a meaningful duration to overcome this hurdle. Studies have shown that SFN is rapidly absorbed and eliminated with minor inter-individual variations, with typical urinary excretion accounting for 70−90 % of the administered dose [30,31].

## 2 Materials and methods

### 2.1 Cells and reagents

The murine macrophage cell line RAW 264.7 (ATCC TIB-71) and the human neuroblastoma cell line SH-SY5Y (ATCC CRL-2266) were obtained from the American Type Culture Collection (ATCC; Manassas, VA, USA) and confirmed to be free of mycoplasma contamination. Both cell lines were screened, verified to be mycoplasma-free, and authenticated via short tandem repeat (STR) analysis six months before the start of this study. The cells were cultured in DMEM media (Corning, USA) containing 10 % fetal bovine serum (FBS; Thermo Fisher Scientific, Waltham, MA, USA), 100 μg/mL penicillin, and 100 μg/mL streptomycin in a humidified atmosphere of 5 % CO_2_ at 37 °C. For SH-SY5Y cell division, 350 μL of the cell suspension was seeded in a 100 mm dish and cultured for 48 h at 37 °C. The cells were allowed to grow until they reached approximately 80 % confluency. To ensure proper cell adhesion to the plates, 100 μL of poly-L-lysine (P-L-L) was added to each well and left for 1 h. After 1 h, the P-L-L was removed, and the plates were left to dry for an additional hour. Subsequently, 100 μL of DMEM containing 10 % FBS was added to each well. The cells were then seeded at a density of 20 μL per well and incubated for 24 h until they reached 80−90 % confluency, at which point they were ready for further experimental procedures. The RAW 264.7 cells were passaged at 60−70 % confluency and changed the media for every 2−3 days for both cells. All the other reagents used are analytical/HPLC grade and cell culture plasticwares from SPL Life Sciences, South Korea. The D,L-sulforaphane HPLC standard (25 mg) was purchased from Merck Millipore, Germany.

### 2.2 Extraction protocol and SFN content analysis

#### 2.2.1 Broccoli sprout extract (BSE) preparation and optimization

Scientifically grown broccoli sprouts were obtained from designated farming sites in Jeollanam-do province, South Korea. The broccoli sprouts underwent harvesting, drying, and pulverization as part of the extraction process to optimize the yield of SFN. Fresh sprouts were meticulously washed with deionized water to eliminate any surface contaminants. Subsequently, the cleaned sprouts were subjected to drying in a mechanical drier for 2−3 days until reaching 70−80 % dryness while retaining their green coloration. Following drying, the sprouts were pulverized and stored in a humid (60 %)- and temperature (20 °C)-controlled environment until further use. This endogenous extraction protocol was developed to enhance the yield and quality of SFN while minimizing environmental impact. Detailed specifications of the extraction protocol are currently withheld due to pending patent applications. The extraction process was fine-tuned to optimize incubation time (1 h) and duration (3 h), aiming to achieve a high content of SFN (460.75 ± 10.27 mg/L) of SFN. The resulting supernatant extract underwent meticulous filtration using Whatman filter paper A. Dissolved sugar content in the BSEP was measured using a refractometer (ATAGO, Japan) against control and result values expressed in Brix (1 Brix = 1 g of sucrose in 100 g of solution). The resultant extract was subjected to analysis to evaluate its antioxidant and anti-inflammatory capacities, as well as its potential applicability in AD treatment.

#### 2.2.2 Quantification of SFN in BSEP using HPLC & mass spectrometry

A 20 μL of 1 % BSEP solution was filtered through a 0.45 μm nylon membrane filter, then the filtrate was injected into the HPLC chromatography (FuTec LC6000, South Korea) equipped with a UV/Vis detector at 25 °C. Chromatographic separation was performed using a ProntoSIL C18 SH column (250 × 4.6 mm, particle size 5 μm, Bischoff Chromatography, Germany). The mobile phase consisted of HPLC-grade water (A) and acetonitrile (B). An isocratic gradient was maintained at 22 % acetonitrile for 20 min. The flow rate was set at 0.8 mL/min, and the column temperature was maintained at 25 °C. SFN was detected by comparing its chromatographic behavior and monitoring UV absorption at 202 nm using authentic standards and reported data.

#### 2.2.3 Calibration of SFN standard curve

Standard stock was obtained at a concentration of 25 mg/mL (Merck Millipore, D,L-SFN) accurately, and then was diluted by addition with a volume of ethanol to approach the working concentrations. The calibration curves were built using four different concentrations for analyte and calculating correlation coefficients (r^2^). In detail, the concentrations were prepared ranging from 25 to 200 ppm. The linearity was conducted for calibration curve at triplicated time in an independent manner, and limit of detection (LOD) value was obtained.

### 2.3 Storage stability evaluation of the BSEP

1 % BSE was lyophilized using a freeze dryer (Ilshin BioBase, Japan) for five consecutive days. The moisture content was measured with an MB25 Moisture Analyzer (Ohaus, USA). In this process, 5 ± 0.013 g of BSEP was placed in a pre-weighed aluminum pan on the moisture analyzer, allowing the water content to evaporate until a stable value was reached. This experiment was repeated three times. For the shelf-life study, 250 mg of BSEP was encapsulated in commercial gelatin capsules. Ten capsules were placed in a 50 mL conical tube (SPL Life Sciences, South Korea). These tubes were stored at 4 °C (refrigerator), 25 °C (temperature-controlled room), and 40 °C (temperature-controlled incubator) for an extended period. One capsule from each storage condition was sampled monthly, and the SFN content was analyzed using the HPLC method from a 1 % solution in water against control. The results were also correlated with the physical appearance of the powder, particularly in terms of color changes.

### 2.4 Antioxidant capacity measurements

The oxygen radical absorbance capacity (ORAC) assay was conducted to evaluate the antioxidant capacity of the BSE using the Zen-Bio ORAC Antioxidant Assay Kit, following the manufacturer's protocol. The assay relies on monitoring the loss of fluorescein fluorescence over time, induced by the generation of peroxyl radicals from the breakdown of AAPH (2,2′-azobis-2-methyl-propanimidamide, dihydrochloride). Trolox [6-hydroxy-2,5,7,8-tetramethylchroman-2-carboxylic acid], a water-soluble vitamin E analogue, served as the standard antioxidant. Before commencing the experiment, the plate reader's incubation chamber was equilibrated to 37 °C. The plate reader was configured to conduct a kinetic read with 1-min intervals over a 30-min duration. A 96-well microplate was prepared by dispensing 25 μL of diluted extracts or Trolox standards into the designated wells. Subsequently, fluorescein (150 μL, 40 nM) was added to each well, and the plate was incubated at 37 °C for 10 min. Following the incubation period, 25 μL of 2,2′-azobis (2-amidinopropane) dihydrochloride (AAPH, 153 mM) was added to initiate the reaction. Fluorescence intensity was measured at excitation and emission wavelengths of 485 nm and 535 nm, respectively, every minute for a duration of 90 min using a microplate reader. The area under the fluorescence decay curve (AUC) was calculated for each sample and standard using appropriate statistical software (OriginPro 2023). The antioxidant capacity was expressed as micromoles of Trolox equivalents (TE) per gram of extract (μmoL TE/g), based on the Trolox standard curve. This ORAC assay protocol ensures precise and reproducible quantification of the antioxidant potential of the extracts, facilitating accurate assessment and comparison of their antioxidant capacities.

### 2.5 Cell viability assay

Quanti-Max™ WST-8 Cell Viability Assay Kit (Biomax, Korea) was used to measure the viability of cells against BSEP solution following the manufacturer's protocol. Concisely, 5000 RAW 264.7 and SH-SY5Y cells were seeded per well of 96-well microtiter plate in 100 μL 1 × DMEM (HG) + 10 % FBS + 1 % antibiotic-antimycotic solution, in separate experiments. The plate was then incubated for 24 h at 37 °C in a 5 % CO_2_ chamber. The next day, the plate confirmed that the cells reached 80−90 % confluency in each well and the media was removed. The cells were washed once with sterile 1 × D-PBS and 100 μL of fresh cell culture media was added. Followed by required dilutions of BSEP to the respective testing wells in triplicates. The plate was incubated for a further 24 h in the incubator. The following day, 10 μL of Quanti-Max™ reagent was added to each well and incubated for a maximum of 1 h at 37 °C in a 5 % CO_2_ chamber. The absorbance was read out at 450 nm in a microplate reader (Tecan Infinity F Nano+) and the results were represented as graphical plots. 100 μL of the culture medium mixed with 10 μL of Quanti-Max™ was used as a blank. (Cell-free culture medium + Quanti-Max™). Teflon and latex were used as the positive and negative controls, respectively.

### 2.6 Anti-inflammatory test

RAW 264.7 cells at passage 3 were seeded at 1 × 10^5^ cells/mL to each well of a 6-well cell culture plate. The cells were cultured until it reached 70−80 % confluency and media were changing every 2 days. Upon confluency, removed the old media and cells were pre-treated with 1 mL non-serum media for 10−15 min in the incubator. All controls and samples, dilutions are prepared in non-serum media. Removed non-serum media and added with LPS (1 μg/mL) in non-serum media for 24 h. Next day, removed the LPS containing media and washed once with 1 × PBS and then the cells were treated with SFN solutions for 1 h. Removed the treatment solution, washed twice with 1 × PBS, pH 7.4. The cells were dissociated and collected by sedimentation (1500 rpm, 6 min).

#### 2.6.1 Gene expression profiling of inflammatory genes

The total RNA content was extracted following the TRIzol method according to the manufacturer's protocol. Quantification of mRNAs abundance was determined using 1-step reverse transcription real-time quantitative PCR with the QuantiTect SYBR Green RT-PCR Kit (Qiagen, Germany), performed on a Rotor-Gene Q Real-Time system (Qiagen, Germany). The gene primers were used respectively from the work of Wang et al., 2008 [32] (IL-6, TNF-α, COX-2, and ACT-β) Each reaction contained 100 ng total RNA, and for gene expression, 0.5 pmoL primers respective primers, 5 μL 1-step reaction mix, and 0.1 μL Quantiscript reverse transcriptase were included in the 10 μL reaction system and ran at 50 °C for 30 min, 95 °C for 15 min, 40 cycles of 94 °C for 15 s, and 52−58 °C for 30 s. Each sample assay was performed in triplicate to determine an average threshold cycle (C_t_) value. Target gene expression was normalized to constitutively expressed ACT-β gene, and the relative quantity of target gene mRNAs was expressed as 2^−ΔΔCt^ using the relative comparative threshold cycle method as previously described [[33], [34], [35]]. For each PCR run, a non-template control and a non-reverse transcriptase control were performed to make sure there was no genomic DNA contamination in the sample or the master mix.

### 2.7 Preparation and induction of AD model

#### 2.7.1 Animals and treatments

**Fig. 1:**
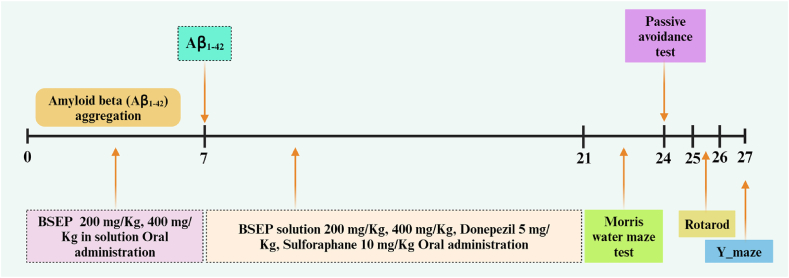
Experimental process to confirm the effectiveness of broccoli sprout extract powder.

Seven-week-old ICR male mice (25−30 g body weight range) were purchased from the Samtaco BIO, KOREA (Osan-si, Gyeonggi-do, Korea). The mice were maintained on a 12-h light–dark cycle with controlled temperature at 22 ± 3 °C, humidity at 50 % + 10 %, and food (Samtaco BIOKOREA, Korea) and water ad libitum for one week. All experimental procedures were conducted in accordance with the relevant guidelines for the care of experimental animals and approved by the Institutional Animal Care and Use Committee (IACUC) of Bioresources and Technology (B&Tech) Co., Ltd. (Gwangju, Republic of Korea; approval no.: BT-001-2023). All efforts were made to minimize suffering and the number of animals used. One day before dissection, only water was provided and food was fasted. Acclimatized experimental animals were grouped into seven containing 7 mice each (Table 1 and Fig. 1).

**Table 1:** Definition of test group.

Group	Administration	Dose (mg/kg)	Route of administration
Normal control	Saline	0	Oral
Sham	Saline	0	Oral
Negative control (AD)	Saline	0	Oral
BSEP (400)	BSEP	400 (equivalent to 10 mg/kg SFN)	Oral
BSEP (200)	BSEP	200 (equivalent to 5 mg/kg SFN)	Oral
Positive control 1 (Donepezil)	Donepezil	5	Oral
Positive control 2 (SFN (10))	SFN	10	Oral

#### 2.7.2 Preparation of test samples

Prepare the test substance at a concentration appropriate for each test using purified water. The control material (Donepezil) was prepared using 0.5 % CMC solution and purified water. Amyloid beta (Aβ_1-42_) was prepared and administered following standard protocols. BSEP was orally administered at doses of 200 mg/kg/day and 400 mg/kg/day for one week before and after Aβ_1-42_ injection, each at a volume of 200 μL. Following intravenous administration, broccoli extract was orally administered at the same doses for two weeks. Additionally, SFN and donepezil were orally administered at doses of 10 mg/kg and 5 mg/kg, respectively, each at a volume of 200 μL, for two weeks.

#### 2.7.3 Alzheimer's animal model induction

Aβ_1-42_ (100 mM) was surgically injected into the brain ventricle of anesthetized mice (intravenous [IV] injection of xylazine [5 mg/kg] and ketamine [50 mg/kg]). The skull was precisely punctured at specific coordinates using a small drill, and amyloid beta was injected deeply into the brain using a microinjector. The control group underwent the same procedure with saline injections instead. Following surgery, the incision sites were disinfected and sutured, and the animals were allowed to recover in a cage.

### 2.8 Brain tissue TUNNEL assay

The mice were anesthetized by an intraperitoneal injection of ketamine (50 mg/kg) (Yuhan, Seoul, Korea) and xylazine (5 mg/kg) (Bayer AG. Leverkusen, Germany) mixture and their whole blood was removed by PBS perfusion at a rate of 2 mL/min for 5 min. Then, brain tissues were fixed with 4 % paraformaldehyde perfusion at a rate of 2 mL/min for 10 min. The brain was collected carefully and stored in 4 % paraformaldehyde solution for 48 h for additional fixation. The solution was sequentially exchanged with 15 % and 30 % sucrose solutions until the tissue sinks. Brain tissue was frontally sliced at the injection point and immediately embedded in OCT compound (Leica Biosystems, Wetzlar, Germany). Tissue slides were made at a 6-μm thickness by cryostat sectioning at −25 °C and stored in freezer for further analysis. Apoptotic cells were determined by an *In-Situ* Cell Death Detection kit (Sigma, St Louis, MO, USA) according to the manufacturer's recommended procedures. The nuclei were counterstained by mounting medium containing DAPI (Thermo Fisher Scientific, Waltham, MA, USA). Apoptotic cells were observed under a fluorescence microscope (Nikon, Tokyo, Japan). The number of apoptotic cells was counted in hippocampal regions of each section, and the average was taken.

### 2.9 Behavioral tests

#### 2.9.1 Morris water maze test

The water maze test evaluates the spatial memory of experimental animals by filling a circular pool (120 cm diameter × 60 cm hight) with water to a depth of 30 cm (maintained at 23 ± 2 °C). An escape zone (10 cm diameter) is designated in one section of the four quadrants, with the platform positioned 1 cm below the water surface and made invisible with skim milk powder. If the animal fails to locate the platform within 60 s, it is gently guided there and allowed to familiarize itself for 20 s. The test is conducted at 30-min intervals, four times daily for three consecutive days. Observations are recorded using a ceiling-mounted camera and analyzed using SMART 3.0 software (Panlab S.L., Barcelona, Spain).

#### 2.9.2 Passive avoidance test

The passive avoidance experiment is a commonly used method for assessing enhancements in learning and memory capabilities. The experiment was conducted in a room with noise levels below 60 dB and dim lighting. The measuring instrument, Shuttle Box Test Package (Med Associates Inc., Vermont, USA), consisting of two rooms of equal size (25 × 25 cm). An automatic guillotine door was installed on the wall separating the two rooms. One room was illuminated (20 W light bulb), while the other remained dark. The mice were trained to associate the dark environment with an aversive stimulus. In the experiment (*n* = 3), upon entry into the dark room, an electric shock (foot-shock) was administered by passing a current of 3 mA for 5 s through a stainless-steel grid beneath the room. This procedure facilitated the mouse's recognition of the association between the dark environment and the aversive stimulus.

#### 2.9.3 Rotarod test

The rotarod test was performed by placing a rat on a rotarod device (Panlab, Harvard apparatus, Massachusetts. USA) and starting at a speed of 4 rpm, gradually increasing the speed to 40 rpm after 300 s, and then rotating the treadmill. When asked to do so, the time (s) until the experimental rat lost its balance and fell to the floor was measured.

#### 2.9.4 Y-maze test

The Y-maze test serves as an experiment to assess the restoration of short-term memory and spatial perception abilities in experimental animals. This test employs a Y-shaped enclosed gray acrylic maze, with each branch positioned at a fixed angle of 120° to one another. The experimental animals are allowed to freely navigate the Y-maze for a duration of 8 min in designated area with activity recorded via SMART 3.0 (Panlab S.L., Barcelona, Spain). Spontaneous alternation is evaluated by monitoring the number and sequence of entries into each branch. An alternation is acknowledged as 1 point (actual alternation) when the experimental animal sequentially enters three distinct branches in the order of ABC, BCA, or CAB. Conversely, no points are awarded if the entries fail to occur consecutively. The alteration rate is computed as the ratio of actual alternations to maximum potential alternations, expressed as a percentage ([actual alternations]/[maximum alternations] × 100).

### 2.10 Brain tissue acetylcholine (ACh) and acetylcholinesterase (AChE) activity

Following the behavioral evaluation test, the experimental animal was euthanized, and its brain was extracted. Brain tissue was homogenized in 700 μL of PBS and centrifuged at 14,000 rpm at 4 °C for 10 min to obtain the supernatant. The supernatant was analyzed for acetylcholine (ACh) and acetylcholinesterase (AChE) activity using specific assay kits (ACh assay kit: ab65345, AChE assay kit: ab138871, Abcam, Cambridge, UK). These assays utilize enzymatic reactions to quantify ACh and AChE levels, providing insights into cholinergic neurotransmitter function.

### 2.11 In vivo oral absorption of SFN and BSE in rats

Sprague–Dawley rats (males, 6–7 weeks old, 200–250 g) were purchased from G-bio (Gwangju, Republic of Korea). The animals were housed under standard conditions in terms of temperature (23 ± 2 °C), relative humidity (RH; 55 ± 10 %), and light (12/12 h light/dark cycle). The animals had ad libitum access to a standard laboratory diet (Nestlé Purina PetCare Research, St. Louis, MO, USA) and ion-sterilized tap water. Ethical approval for this study was obtained from the IACUC of Mokpo National University (Jeonnam, Republic of Korea; approval no. MNU-IACUC-2023-009).

To examine the distinction of pharmacokinetic parameters of SFN after IV injection and oral administration of SFN or BSEP (containing 3 % SFN), Sprague–Dawley rats were orally administered 400 μL of SFN dissolved in 0.5 % sodium CMC (5 mg/kg, SFN-oral) or aqueous solution of BSEP (equivalent to 5 mg/kg SFN, BSEP-oral). To calculate the oral bioavailability, rats were intravenously injected with 200 μL of SFN (5 mg/kg SFN dissolved in 0.5 % sodium CMC) via the femoral vein. Then, 200-μL blood samples were collected from the cannulated femoral artery at predetermined time points and immediately centrifuged at 13,000×*g* for 5 min at 4 °C. The separated plasma samples were kept frozen at −80 °C until analysis.

To determine the plasma concentrations of SFN, the working solutions of SFN of 20, 15, 10, 5, 2, 1, 0.6, and 0.2 μg/mL, and 5 μg/mL phenylethyl isothiocyanate (internal standard, IS) were prepared in acetonitrile. Then, 10 μL of each working solution and 10 μL of IS were spiked with 180 μL of rat blank plasma for standard solution. Separately, 180 μL of each plasma sample was spiked with 10 μL of IS solution and 10 μL of acetonitrile. Next, 200 μL of a mixture of acetonitrile, water, and ethanol (25:25:50, v/v/v) was added to each plasma sample spiked with IS, followed by vortex mixing. After centrifugation at 13,000×*g* for 5 min at room temperature, the supernatant was transferred to another vial for further extraction using an HLB SPF μElution plate (Waters Corp., Milford, MA, USA). Each well of the SPE plate was conditioned and equilibrated by 200 μL of methanol and water, respectively. Then, the 400 μL of the collected supernatant was added to each well of the plate and aspirated under a 5 H g vacuum. After washing with 200 μL of water and 50 % methanol, each well was eluted with 400 μL (200 μL × 2 times) of acetonitrile.

Each eluted sample (10 μL) was injected into a UPLC system (ACQUITY UPLC; Waters Corp.) equipped with an ACQUITY UPLC BEH C18 column (2.1 × 50 mm, 1.7 μm; Waters Corp.) at 50 °C. SFN and IS were eluted by the mobile phase (10 mM ammonium acetate in water:0.1 % formic acid in acetonitrile [3:7, v/v]) at a flow rate of 0.6 mL/min. A mass spectrometer (Xevo TQ-S; Waters Corp.) was used for ionization of SFN or the IS (in positive ion multiple reaction-monitoring mode) at an electrospray voltage of 3.0 kV, with a source temperature of 120 °C, desolvation temperature of 500 °C, and desolvation gas flow rate of 800 L/h. SFN quantification was performed by reference to the *m/z* transition 178.0 → 114.0 at a cone voltage of 24 V and collision energy of 30 eV; for IS, the *m/z* transition was 164.0 → 130.0 at a cone voltage of 23 V and collision energy of 22 eV.

### 2.12 Statistical processing

Statistical analysis was conducted utilizing the OriginPro 2023 and GraphPad Prism 8.0 software, and experimental results were presented as mean ± standard deviation (SD). Significance was assessed and tested at the p < 0.05 level, indicating statistical significance.

## 3 Results

### 3.1 SFN calibration curve and mass spectrometry confirmation

**Fig. 2:**
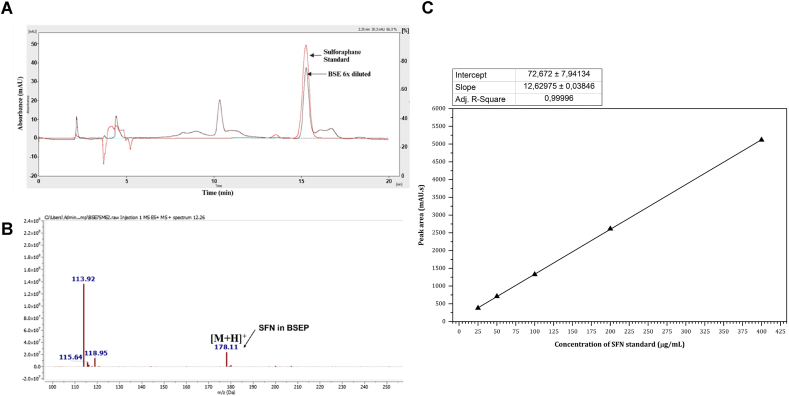
HPLC and LC/MS analysis of BSE. (A) Chromatogram showing the SFN peak in BSEP sample (Black color, labelled) against the SFN HPLC standard (Red color, labelled). (B) Liquid chromatography-mass spectrum of BSEP sample [M+H]^+^ showing the detection spectrum of molecular weight 178.11. (C) Calibration curve of pure SFN using the optimized protocol (*n* = 4, r^2^ = 0.9997).

SFN was identified based on its retention behavior compared to an authentic standard and further confirmed by its *m*/*z* value (Fig. 2A and B). The retention time of SFN was 15.262 min. To determine the calibration curve of SFN, standard solutions were analyzed by LC/MS. The peak area (Y) of SFN was measured and plotted against its concentration (X). The regression equation obtained was Y = 12.998X + 66.58 (*n* = 4). A strong linearity was observed between peak areas and concentrations in the range of 25–200 μg/mL, with a correlation coefficient of 0.9997 with LOD of 25 ppm (Fig. 2C).

### 3.2 Endogenous extraction protocol

Due to an ongoing patent application, we are unable to disclose all the data pertaining to the optimized extraction protocol in the results section. However, we will provide key insights and an overview of the findings to highlight the significance and effectiveness of our approach. Based on the experimental results, it was found that endogenous extraction resulted in the highest SFN content in the extract (460.75 ± 10.27 ppm). Conversely, at higher temperatures, the SFN content decreased to 405.41 ± 67.98 ppm, representing a 12 % reduction even more decline with increasing temperature. Extraction conducted at lower temperature compared to the optimized temperature yielded the moderate but good SFN content (435.24 ± 1.09 ppm). Notably, the most substantial SFN content, approximately 300 ppm, was achieved after the incubation in a water bath. Subsequent hours of incubation led to a significant decrease in SFN content, with a considerable reduction compared to the initial value. The trend shows a decline in SFN content in longer incubations, albeit with a relatively minor margin of decrease.

### 3.3 Moisture content and storage stability of BSEP

**Fig. 3:**
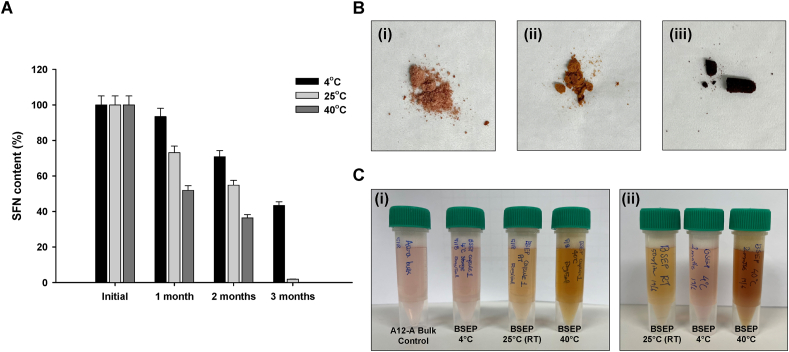
Storage stability of BSEP. (A) SFN content in BSEP stored at 4 °C, 25 °C, and 40 °C over 3 months. Data are expressed as mean ± SD (*n* = 3). (B) The color and physical appearance of BSEP after storage at (i) 4 °C, (ii) 25 °C, and (iii) 40 °C for 3 months. (C) Comparison of solubility and solution color of BSEP stored at 4 °C, 25 °C, and 40 °C for (i) 1 month and (ii) 3 months, with the control shown as the first tube in (i).

The average moisture content of BSEP was 3.25 ± 0.52 %. No significant difference in SFN content in BSEP stored at 4 °C (339.29 ppm) compared to control (363.26) within 30-days storage (Fig. 3A). Whereas 29.23 % and 56.68 % reduction in SFN content was noted in 4 °C storage at the end of 60- and 90-days storage. In contrast, SFN was drastically reduced in 25 °C (26.86 %) and 40 °C (48.10 %) temperatures within 30 days of storage. More than 95 % of the SFN was degraded at these temperatures after 90 days storage. In case of color of BSEP, refrigerated storage retain the pinkish-brown color as similar to control, whereas it turned light brown and dark brown appearance at 25 °C and 40 °C, respectively, in 90-days storage. Additionally, the powder exhibited increased adhesiveness and cohesion (Fig. 3B and C).

### 3.4 Antioxidant capacity of BSE expressed as ORAC value

The ORAC assay was employed to assess the antioxidant capacity of the BSE, revealing significant antioxidant activity across all samples. The ORAC values ranged from 5 to 8 mmoL TE/g, indicative of a high level of antioxidant potential within the extracts. Notably, at the same concentration level (1 mM), the antioxidant activity of the BSEP solution was approximately four times that of pure SFN and 6.6 times that of Trolox. This enhanced activity may be attributed to the combined effect of other isothiocyanates and glucoraphanin present in the extract, as evidenced by the ORAC value of 1716.52 observed for BSEP at 1 %. Therefore, the approximate SFN effect in the BSEP solution was calculated to be 6083.48 μmoL TE/g. The standard curve generated with Trolox exhibited a linear relationship (r^2^ = 0.98), validating the accuracy of the assay. Furthermore, the extracts consistently demonstrated reproducible ORAC values across multiple replicates, affirming their potent antioxidant properties.

### 3.5 The effect of BSEP on the cell viability of macrophages and neuronal cells

**Fig. 4:**
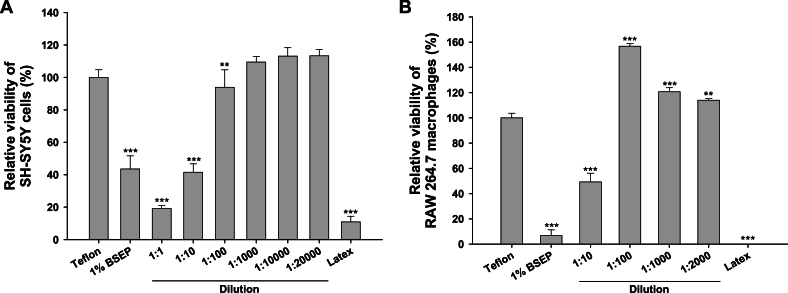
Effect of BSEP on the viability of (A) SH-SY5Y neuronal cells and (B) RAW 264.7 macrophages. Data are expressed as mean ± SD (*n* = 4). ∗∗p < 0.01, ∗∗∗p < 0.001 compared to the control (Teflon).

The impact of BSEP on the viability of RAW 264.7 cells and SH-SY5Y cells was assessed utilizing the WST assay. The cells were exposed to varying concentrations of 1 % BSEP, ranging from a 1:1 dilution to a 1:20000 dilution (a serial dilution of 2 μg/mL to 1 × 10^−4^ μg/mL in 10-fold dilution with non-serum media), for 24 h alongside control groups. The findings revealed a dilution-dependent effect of 1 % BSEP on the viability of both RAW264.7 and SH-SY5Y cells (Fig. 4). In both instances, dilutions from 1:100 onwards exhibited comparable viability to the control group, with minimal to no observed toxic effects. Conversely, direct application of undiluted 1 % BSEP to RAW 264.7 cells resulted in only 6.83 % viability. The outcome differed slightly in SH-SY5Y cells, where lower dilutions such as 1:1 and 1:10 caused a 50 % reduction in cell viability. Results were juxtaposed with those obtained from Teflon and latex controls.

### 3.6 Anti-inflammatory effect of BSEP

**Fig. 5:**
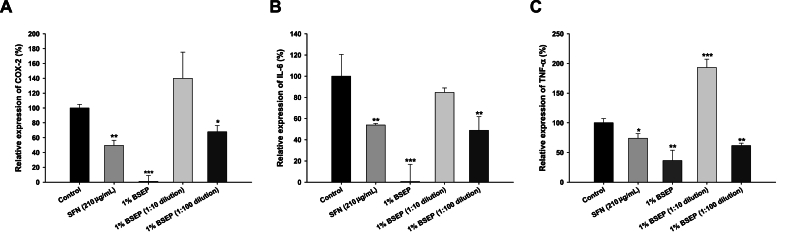
Inhibition of BSEP in the LPS-induced inflammatory response of RAW 264.7 macrophages. Gene expression profiles of (A) COX-2, (B) IL-6, and (C) TNF-α. Data are presented as mean ± SD (*n* = 4). ∗p < 0.05, ∗∗p < 0.01, ∗∗∗p < 0.001 compared to the untreated control.

The gene expression levels of pro-inflammatory cytokines, including IL-6, COX-2, and TNF-α, in LPS-stimulated macrophages (RAW 264.7 cells) were assessed using quantitative PCR. Experimental data indicated a significant downregulation of TNF-α in both the undiluted positive control (210 ppm) and the undiluted 1 % BSEP solution (Fig. 5). However, when comparing the 1:100 dilution of the positive control and the 1:100 dilution of the 1 % BSEP-treated samples with the control (LPS stimulation only), TNF-α expression was notably reduced by approximately 30–40 %, with the 1 % BSEP 1:100 dilution exhibiting a more pronounced effect (12 % greater reduction). A similar trend was observed in IL-6 expression. In contrast, COX-2 showed an 18 % greater reduction in the 1:100 dilution of the 1 % BSEP. This suggests that SFN in BSEP possesses anti-inflammatory properties. However, surprisingly, the nullification of the anti-inflammatory effect was observed in the 1:10 dilution of 1 % BSEP, leading to an upregulated expression profile of all three genes (Fig. 5). Regarding COX-2 expression, 1 % BSEP at 1:100 dilution showed significantly lower expression compared to the control, but slightly higher compared to the SFN standard at the same concentration (Fig. 5). This may be attributed to COX-2 expression being primarily dependent on IL-1β stimulation, along with co-stimulation from other ILs.

### 3.7 Neuroprotective effect of BSEP

**Fig. 6:**
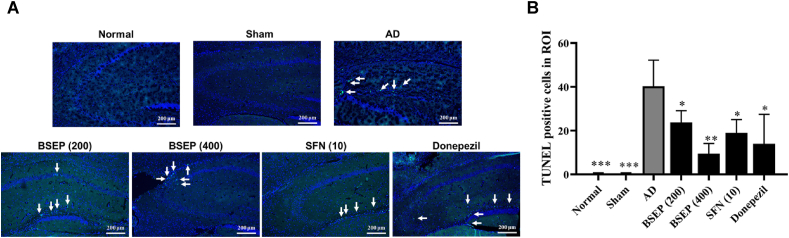
Effect of broccoli extract on hippocampal apoptosis induced by amyloid beta injection. (A) Representative images of each group showed TUNEL positive cells in hippocampal region which were acquired at 100× magnification. (B) The apoptotic cells were counted from fluorescent microscopic images. Data are presented as the mean ± SD (*n* = 4). ∗p < 0.05, ∗∗p < 0.01, ∗∗∗p < 0.001 compared to the AD group.

Apoptotic cells were increased in amyloid beta injected animals whereas they were not found in mice received sham operation. The apoptotic cells were mainly found in CA3 and DG zones, and rarely found in CA1 zone (Fig. 6A). The number of TUNEL-positive cells in the hippocampus was almost 40.3 ± 10.4 cells in the AD group. but decreased to 23.8 ± 4.7 and 9.5 ± 4.0 cells in the groups administered 200 and 400 mg/kg of BSEP, respectively (Fig. 6B). SFN and donepezil received groups showed 19.0 ± 5.2 and 14.0 ± 11.6 cells, respectively. Therefore, this result demonstrated that broccoli extract has neuroprotective effect against amyloid beta neurotoxicity.

### 3.8 Behavioral tests

#### 3.8.1 Water maze experiment

**Fig. 7:**
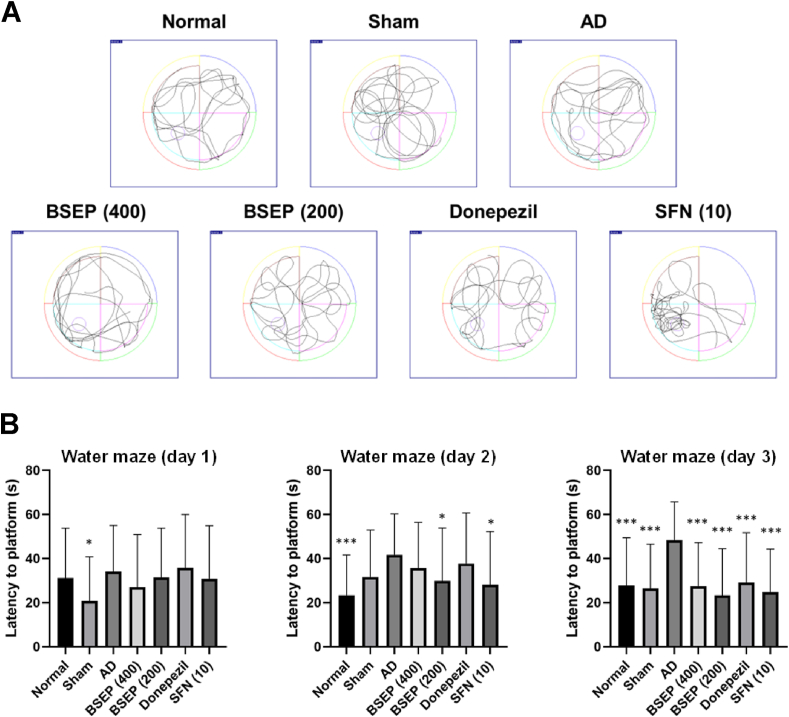
Effect of BSE on spatial learning and memory impairment in AD-lesioned mice (Morris water maze test). (A) Representative swimming trajectories during Morris water maze experimental training. (B) Effect of BSEP solution on Morris water maze experiment in memory-impaired mice using amyloid beta (Aβ_1-42_). Data are presented as the mean ± SD (*n* = 7). ∗p < 0.05, ∗∗∗p < 0.001 compared to the AD group.

The impact of BSEP solution on spatial learning in mice, induced with memory impairment by Aβ_1-42_ injection, was assessed via a water maze experiment, aimed at evaluating hippocampus-dependent spatial learning ability. Following cognitive training conducted four times daily for three consecutive days, the control group exhibited no reduction in escape latency time to locate the platform in the water maze, consistent with findings from numerous dementia model experiments involving Aβ_1-42_-induced memory damage (Fig. 7A). This confirmed the successful induction of long-term memory impairment. Conversely, memory impairment induced by Aβ_1-42_ was significantly ameliorated in the normal control group, sham group, positive control group 1 (donepezil), positive control group 2 (SFN (10)), experimental group 1 (BSEP (400)), and experimental group 2 (BSEP (200)) from the second day of cognitive training (p < 0.05). By the third day, the sham group, donepezil, SFN (10), BSEP (400), and BSEP (200) exhibited escape latency times comparable to those of the normal control group, demonstrating significant improvement compared to the AD group (Fig. 7B).

The average escape latency time over the three days was 42.7 ± 9.13 s for the AD group, significantly longer than that of the other experimental groups, namely the normal group (27.66 ± 3.97 s), sham group (26.3 ± 5.45 s), donepezil (34.97 ± 3.21 s), SFN (10) (28.01 ± 2.95 s), BSEP (400) (31.05 ± 4.36 s), and BSEP (200) (29.91 ± 1.54 s) (Fig. 7B). These results suggest that broccoli extract may effectively enhance long-term memory in mice with dementia induced by Aβ_1-42_.

#### 3.8.2 Passive avoidance test

**Fig. 8:**
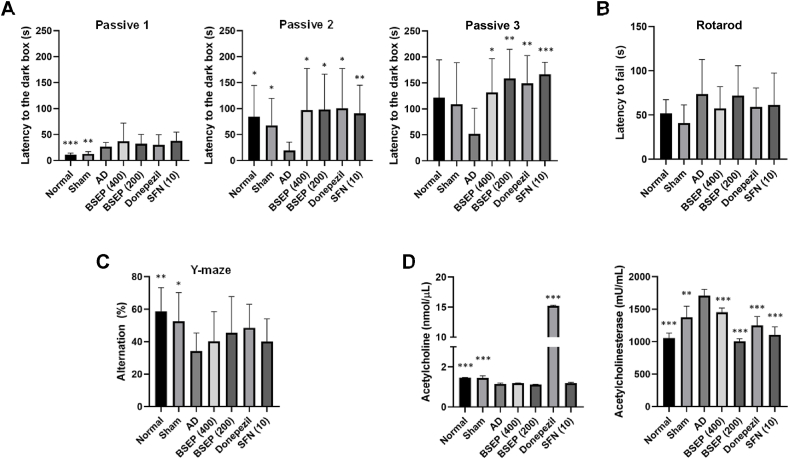
Effect of BSE on spatial learning and memory impairment in AD-lesioned mice (Passive avoidance test; Y-Maze test; Rotarod test & AChE activity). (A) Effect of BSEP solution on passive avoidance test in memory-impaired mice using amyloid beta (Aβ_1-42_). (B) Effect of BSEP solution on rotarod test in memory-impaired mice using amyloid beta (Aβ_1-42_). (C) Effect of BSEP solution on Y-maze test in memory-impaired mice using amyloid beta (Aβ_1-42_). (D) Effect of BSEP solution on ACh content and AChE activity in mice with memory impairment using amyloid beta (Aβ_1-42_). Data are presented as the mean ± SD (*n* = 7). ∗p < 0.05, ∗∗p < 0.01, ∗∗∗p < 0.001 compared to the AD group.

In the passive avoidance experiment, mice typically exhibit a tendency to explore new environments, with a preference for darker areas. This experiment serves to gauge the duration of memory retention for unpleasant stimuli and primarily assesses learning memory. In the control group, the time taken to transition from a brightly lit room to a darkened room increased to 25, 19, and 51 s, while in the normal group, it increased to 11, 84, and 121 s (Fig. 8A). Comparatively, the AD control group displayed a decline in cognition and memory, spending less time in the brightly lit room compared to the normal group. Conversely, the sham group (12, 67, and 108 s), donepezil (29, 100, and 149 s), SFN (10) (37, 90, and 166 s), BSEP (400) (36, 97, and 131 s), and BSEP (200) (32, 98, and 158 s) exhibited a significant increase in the time spent in the brightly lit room. This indicates that the AD control group exhibited deficits in memory and learning regarding aversive stimuli compared to the other control groups.

#### 3.8.3 Rotarod experiments

The Rotarod test serves as a valuable neurobehavioral assessment tool for evaluating balance, limb coordination, and muscle coordination following surgery. Upon measuring the duration of time spent falling from the treadmill, no statistically significant differences were observed between the AD control group and the other experimental groups (Fig. 8B). This suggests that the surgery-induced damage to the scalp and brain skeleton did not exert a discernible impact on motor skills and neural behavior.

#### 3.8.4 Y-maze test

The results of the Y-maze test, as depicted in Fig. 8, aimed to validate the efficacy of BSEP in enhancing spatial learning and short-term memory in mice with memory deficits induced by Aβ_1-42_ administration. The spontaneous behavioral alteration capacity of the normal group was measured at 58.7 ± 14.5 %, while the AD control group, afflicted with memory impairment due to Aβ_1-42_ administration, exhibited a notably lower rate of 34.3 ± 11 %, representing a significant decrease of 58.4 % compared to the normal group (p < 0.05) (Fig. 8C). This indicates the successful establishment of a model for memory impairment induced by Aβ_1-42_, with evident short-term memory deficits. In BSEP (400) and BSEP (200), administered with 400 mg/kg and 200 mg/kg of BSEP, respectively, the spontaneous behavioral alteration rates were measured at 40.2 ± 18.2 % and 45.5 ± 22.2 %, respectively. Notably, these groups exhibited a recovery to 68.5 % and 77.6 % of the normal group, suggesting a memory-enhancing effect of broccoli extract.

### 3.9 Brain tissue ACh and AChE activity

ACh is a neurotransmitter present in all nerve cells and is closely associated with the cholinergic system of the central nervous system. It is synthesized through the enzymatic action of acetyl CoA and choline acetyltransferase (ChAT) and is subsequently broken down into acetate and choline by the action of AChE. In both experimental animal and clinical studies, the deterioration of the cholinergic nervous system is recognized as a significant factor contributing to the early stages of AD. The impact of BSEP on ACh production is illustrated in Fig. 8D. The ACh levels were as follows: normal group: 1.5 ± 0.0 nmoL/μL, sham group: 1.4 ± 0.1 nmoL/μL, AD control group: 1.1 ± 0.1 nmoL/μL, BSEP (400): 1.2 ± 0.0 nmoL/μL, BSEP (200): 1.1 ± 0.0 nmoL/μL, donepezil: 15.2 ± 0.1 nmoL/μL, SFN (10): 1.2 ± 0.1 nmoL/μL. No significant difference was observed between the control group and the experimental group. When evaluating the effect of broccoli extract on inhibiting AChE activity, the AChE activity of the AD control group was found to be 1707.1 ± 97.12 mU/mL (162 %), representing a 62 % increase compared to the normal group (1057.44 ± 75.41 mU/mL: 100 %). The sham group (1374.7 ± 170.39 mU/mL: 131 %), BSEP (400) (1454.76 ± 63.01 mU/mL: 137 %), BSEP (200) (1006.54 ± 39.84 mU/mL: 95 %), donepezil (1248.51 ± 138.72 mU/mL: 117 %), and SFN (10) (1104.65 ± 123.41 mU/mL: 105 %) exhibited AChE activity suppression levels similar to or lower than that of the normal group compared to the AD control group. Based on these results, it appears that BSEP does not affect the concentration of ACh, but it is effective in improving memory and learning by inhibiting the activity of AChE.

### 3.10 In vivo oral absorption of SFN and BSE in rats

**Fig. 9:**
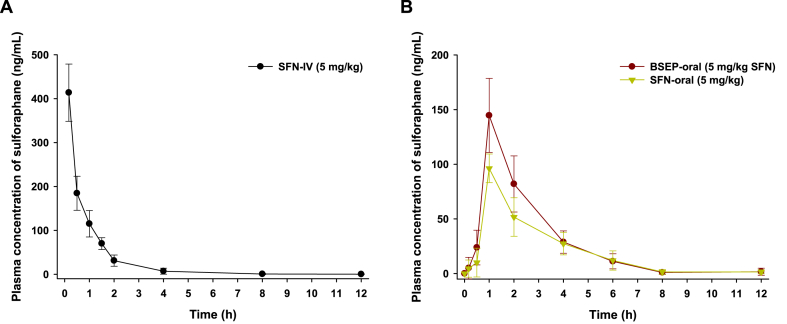
Venous plasma concentration–time profiles of SFN after a single (A) IV dose (5 mg/kg) and (B) oral administration of SFN in 0.5 % sodium CMC (SFN-oral, 5 mg/kg) and BSEP in aqueous solution equivalent to 5 mg/kg SFN (BSEP-oral) to rats. Each value is mean ± SD (*n* = 4/group). SFN, sulforaphane; IV, intravenous; CMC; carboxymethylcellulose; SFN-oral, SFN dissolved in 0.5 % sodium CMC; BSEP, broccoli sprout extract powder.

The comparative mean plasma concentration-time profiles after a single IV dose of SFN (SFN-IV, 5 mg/kg SFN), oral administration of SFN in 0.5 % sodium CMC solution (SFN-oral, 5 mg/kg), or BSEP in aqueous solution (equivalent to 5 mg/kg SFN, BSEP-oral) are illustrated in Fig. 9; the pharmacokinetic parameters are summarized in Table 2. The plasma concentration of SFN increased rapidly after oral administration of SFN-oral (5 mg/kg) or BSEP-oral (equivalent to 5 mg/kg SFN); it reached its maximum at 1 h after intake, indicating rapid absorption. After a single oral dose of SFN was observed in the oral bioavailability to 63.4 ± 12.1 %. On the other hand, the maximum plasma concentration (C_max_) and area under the plasma concentration–time curve (AUC) after oral administration of BSE (equivalent to 5 mg/kg SFN) were 145 ± 33.9 ng/mL and 326 ± 71.4 ng h/mL, respectively; these values were 1.50- and 1.35-fold higher, respectively, than those of SFN-oral (5 mg/kg), demonstrating 1.35-fold greater oral bioavailability (85.7 ± 18.8 %).

**Table 2:** Pharmacokinetic parameters of oral BSEP.

Test material	SFN-IV	SFN-oral	BSEP-oral
Administration route	IV	Oral	Oral
Dose of SFN (mg/kg)	5	5	5
T_max_ (h)	0.170 ± 0.000	1.00 ± 0.000	1.00 ± 0.000
T_1/2_ (h)	1.35 ± 0.806	1.82 ± 0.574	1.28 ± 0.632
C_max_ (ng/mL)	414 ± 65.1	96.2 ± 13.1	145 ± 33.9
AUC_last_ (ng·h/mL)	380 ± 27.5	241 ± 46.1	326 ± 71.4
AUC_inf_ (ng·h/mL)	390 ± 33.7	259 ± 39.6	335 ± 73.8
MRT (h)	0.971 ± 0.224	3.14 ± 0.835	2.55 ± 0.655
Bioavailability (%)	100	63.4 ± 12.1	85.7 ± 18.8

**Notes:** Data are presented as means ± SDs (*n* = 4).

**Abbreviations:** IV, intravenous; T_max_, time of maximum concentration; T_1/2_, half-life; C_max_, maximum concentration; AUC_last_, area under the curve from zero to the observed last time point; AUC_inf_, area under the curve from zero to infinity; MRT, mean residence time.

## 4 Discussion

The experimental results revealed that at the optimal extraction temperature (not revealed due to patent application), the highest SFN content in BSEP yielding an SFN concentration of 460.75 ± 10.27 ppm. This temperature strikes a delicate balance between maximizing yield and maintaining SFN integrity. Generally, the average content of SFN in broccoli is 0.14–370.3 mg/100 g FW [23]. Increasing the temperature resulted in a 12 % reduction in SFN content, but the purity observed at this temperature still makes it a favorable choice. Higher temperatures were detrimental, showing a sharp decline in SFN content [25], while extraction 10 °C below the optimum was less effective, though yielding a significant SFN content (435.24 ± 1.09 ppm). The notable reduction in SFN content in the endogenous extraction compared to the short-term incubations further underscores the critical influence of both incubation temperature and duration on the efficiency of SFN extraction [26]. Interestingly, a substantial SFN content of approximately 300 ppm was achieved after such short-term incubation. In contrast, solvent extraction methods utilize an hour even though a longer duration is necessary for maximum bioactive compound extraction [36]. However, prolonged incubation beyond this period led to a marked decrease in SFN content in endogenous extraction, indicating the importance of optimizing both the temperature and duration.

Compared to our technology, many researchers have utilized conventional multi-step liquid-liquid extraction processes involving organic solvents [23,37,38] or exogenous extraction techniques employing externally added enzymes such as myrosinase [39], salting-out assisted hydrophobic deep eutectic solvents [40], and high-pressure processing [41,42]. Several studies have reported the extraction of SFN from broccoli under conditions of 35–45 ± 2 °C for 1–4 h, typically using organic solvents such as dichloromethane and methyl-t-butyl ether [36,[43], [44], [45]]. In contrast, our endogenous extraction method achieved an SFN content of 86.6 μmoL/g FW, which is over 10 times higher than that obtained through conventional endogenous extraction methods (2.15−8.5 μmoL/g FW) [46]. Moreover, extending the incubation time beyond the optimal period led to a slight decrease in SFN content, indicating that our method can significantly reduce costs by 90 % or more compared to existing methods. Notably, our technology operates at a reasonable temperature and does not rely on toxic organic solvents, making it a faster and more environmentally friendly alternative. This study underscores the importance of optimizing extraction parameters to maximize SFN yield and quality, thereby enhancing the potential health benefits of BSE.

The findings indicate that SFN stability in BSEP is significantly influenced by storage temperature. In this case, refrigeration at 4 °C effectively preserves SFN content for up to 30 days, but prolonged storage leads to notable degradation. Higher temperatures (25 °C and 40 °C) accelerate SFN degradation, with over 95 % loss within 90 days, highlighting the critical importance of low-temperature storage for maintaining SFN potency. Our results were in par with reports of Wu et al. [47] indicated that SFN in broccoli extract is unstable at high temperature. However, if the pH was reduced the SFN could be heat-stable [47,48]. Additionally, the change in color and increased adhesiveness of BSEP at elevated temperatures suggest that higher storage temperatures negatively impact both the chemical and physical properties of the extract. These findings underscore the importance of maintaining controlled storage conditions to preserve the therapeutic efficacy and quality of BSEP. However, further studies are necessary to evaluate the long-term stability of BSEP under various storage environments.

The antioxidant activity of the BSEP was measured as ORAC value. The ORAC assay demonstrates the significant antioxidant capacity of BSEs, with values ranging from 5 to 8 mmoL TE/g. The enhanced antioxidant activity of BSEP, approximately four times that of pure SFN and 6.6 times that of Trolox, suggests a synergistic effect from other isothiocyanates and glucoraphanin present in the extract [[49], [50], [51]]. This combined effect influenced by high phenolic content accentuates the superior efficacy of BSEP in neutralizing free radicals, as reflected by the calculated SFN effect of 6083.48 μmoL TE/g. In another study, ethyl acetate fraction of BSE results a 11-fold higher ORAC value than the distilled water fraction [52]. This might be due to their simple procedure and initial use of 80 % methanol for extraction. It is well documented that hydrophilic extract (65.8–121.6 μmoL TE/g) have high ORAC value than lipophilic extraction (3.9–17.5 μmoL TE/g) [49]. So, a high ORAC value for our extract is not surprising. The high reproducibility of ORAC values across multiple replicates validates the consistency and reliability of the extracts' antioxidant properties. These findings highlight the substantial health-promoting potential of BSEs, particularly in oxidative stress-related conditions.

The experimental findings elucidated SFN's anti-inflammatory efficacy in BSEP by attenuating the inflammatory response in RAW 264.7 macrophage cells. In which, TNF-α expression was significantly downregulated in samples treated with SFN at 210 ppm and undiluted 1 % BSEP solution, likely due to their negative effects on cell viability. Notably, the 1 % BSEP at 1:100 dilution exhibited a more pronounced reduction in TNFα expression compared to the 1:100 dilution of SFN 210 ppm against the LPS-stimulated control, indicating potent anti-inflammatory activity. However, this effect was diminished at a 1:10 dilution, suggesting that excessive dilution could nullify the anti-inflammatory benefits. IL-6 mRNA levels followed similar trends, further supporting the anti-inflammatory potential of BSEP. The current observations were also underlining that SFN stimulation act as an inductor of IL-6 expression targeting Nrf2 activation [53]. In terms of COX-2 expression, the 1 % BSEP at 1:100 dilution showed significantly lower expression compared to the control, albeit slightly higher than the SFN standard at the same concentration, possibly due to the role of co-stimulatory cytokines like IL-1β. The noted expression profile of the pro-inflammatory genes was also consistent with the existing reports of SFN treatment on LPS-induced RAW 264.7 macrophages [[54], [55], [56]]. In contrast, they have all used 70 % ethanolic extract of the broccoli [56]. Similarly, SFN can act as an inhibitor of NF-κβ activity thereby effectively showing anti-inflammatory responses [57,58]. The findings reinforce the efficacy of SFN in BSEP in mitigating inflammation, provided appropriate concentrations are maintained.

Neurological disorders such as Alzheimer's, Parkinson's, Huntington's, multiple sclerosis, amyotrophic lateral sclerosis, traumatic brain injury, spinal cord injury, and cerebral ischemia/reperfusion are highly prevalent across the globe. The underlying causes remain uncertain, but oxidative stress is suspected to be a major factor in their progression [59]. SFN has demonstrated neuroprotective effects on both *in vitro* and *in vivo* models of neurodegeneration [29,60,61]. Notably, isothiocyanates (ITCs), including SFN, are believed to be particularly effective due to their activation of the Nrf2/ARE pathway, which enhances antioxidant defenses [59,62]. We evaluated the neuroprotective and anti-Alzheimer's activity of the endogenously extracted BSE through cell viability assay, documented behavior tests (water maze test, Rotarod test, manual avoidance test and Y-maze test) and brain tissue ACh and AChE activity. The TUNNEL assay revealed a significant increase in apoptotic cells in the hippocampus of amyloid beta-injected mice, particularly in the CA3 and DG zones, compared to sham-operated mice. Treatment with BSEP at doses of 200 and 400 mg/kg significantly reduced the number of TUNNEL-positive cells, as did treatments with SFN and donepezil, indicating that broccoli extract possesses neuroprotective effects against amyloid beta-induced neurotoxicity. This is attributed to its ability to target multiple cellular mechanisms and pathways. We should also consider that the SFN and its precursor glucoraphanin have anti-depressant effects reported in a mice model where repeated SFN treatment significantly decreased immobility time [63,64]. On top of that, BSE showed no negative effects on SH-SY5Y cells in 1:100 dilution treatment in the present study [65].

A crucial aspect of AD pathogenesis is the aggregation of amyloid beta 1–40 peptide (Aβ) from its soluble form in the brain [66,67]. Research indicates that oxidative stress and the abnormal accumulation of Aβ amplify intracellular oxidative damage and trigger an inflammatory response [68,69]. The impact of BSEP solution on spatial learning in mice, induced with memory impairment by Aβ_1-42_ injection, was assessed via a water maze experiment. We aimed at evaluating hippocampus-dependent spatial learning ability. Over three days of cognitive training, the control group showed no reduction in escape latency, confirming successful induction of long-term memory impairment. In contrast, the sham, positive controls, and experimental groups showed significant improvement in escape latency by the second day (p < 0.05), with times comparable to the normal group by the third day. The control group had an average escape latency of 42.7 ± 9.13 s, significantly longer than the other groups, suggesting that broccoli extract may effectively enhance long-term memory in this model of dementia. Similarly, in a study, AD mice had significantly prolonged escape latency from day 1 to day 4 (p < 0.05) against control, but this increase was not significant in SFN-treated AD mice (p > 0.05). The mice also showed significantly shorter escape latencies from day 2 to day 4 and more passing times in the probe trial compared to untreated AD mice (p < 0.05) [60]. In fact. the same results already been documented in mice as SFN has alleviated LPS-induced spatial learning and memory dysfunction [70]. This could be achieved because the SFN has the relation to the regulation of hippocampal brain-derived neurotropic factor (BDNF)-mammalian target of rapamycin (mTOR) signaling pathway [70,71].

Our results were also substantiated by Madhukar and Ashwlayan [72], where 40 and 80 mg/kg orally administered Broccoli flower hydro-alcoholic extract relieved memory deficits and oxidative stress induced by scopolamine. The anti-dementic activity of the extract was detailed with similar observations in water maze test and elevated plus maze test [72]. In the Y-maze test, the control group, afflicted with Aβ_1-42_-induced memory impairment, showed a significant decrease in spontaneous behavioral alteration compared to the normal group 1 (p < 0.05), indicating successful model establishment (Fig. 6). However, experimental group 1 (400 mg/kg BSEP) and experimental group 2 (200 mg/kg BSEP) exhibited improved spontaneous alteration rates, suggesting a potential memory-enhancing effect. Previously, reported that SFN enhanced spatial working memory and contextual memory in Y-maze and passive avoidance tests, despite not directly interacting with Aβ [73]. Although the exact mechanism of SFN in AD remains unclear, the study suggests that SFN can mitigate cognitive impairment and protect the brain from amyloidogenic damage.

In our manual avoidance test study, the control group demonstrated a notable decrease in time spent transitioning from a brightly lit room to a darkened room (25, 19, and 51 s), compared to the normal group (11, 84, and 121 s) (Fig. 8A). This decline suggests impaired cognition and memory retention in the control group relative to the normal group. Conversely, all the other groups also exhibited significant increases in time spent in the brightly lit room, indicating intact memory and learning abilities compared to the control group. Thus, SFN treatment can ameliorate the cognitive function due to Aβ-induced AD [73]. The Rotarod test showed no statistically significant differences in the duration of time spent falling from the treadmill between the control group and the other experimental groups (Fig. 8B), indicating that surgery-induced damage to the scalp and brain skeleton did not affect motor skills and neural behavior [61]. BSEP does not significantly alter ACh concentration, but it effectively inhibits AChE activity, thereby potentially enhancing memory and learning. This suggests that BSEP could be beneficial in mitigating the cholinergic deficits associated with early stages of AD through AChE inhibition. In turn, this could support the anti-dementic effect mediated through the inhibition of AChE activity [72,74,75]. Therefore, BSE through the endogenous extraction containing sufficient amount of SFN level could potentially alleviate the AD-induced lesions. Even though its molecular crosstalk has not been completely elucidated, BSE can be sourced as a pharmacological or functional food to treat or prevent neurodegenerative diseases.

SFN was well-absorbed and rapidly exhibits an absolute oral bioavailability of 63.4 %. In comparison, BSE showed 1.35 times greater oral bioavailability, reaching 85.7 %. Previous studies have reported that BSE has almost equal oral bioavailability to SFN [76]. This increased bioavailability may be attributed to the improved aqueous solubility and lipophilicity of SFN when combined with other natural components in the BSE. Additionally, in rodents, SFN brain concentrations have been reported to reach approximately 0.5–2 μM within a few hours post-administration, depending on the dosage and the route of administration (oral or intraperitoneal) [77,78]. While human studies on SFN brain concentrations are limited, SFN has been detected in human plasma at nanomolar to low micromolar levels following the consumption of broccoli sprouts or supplements [79]. Since SFN can cross the blood-brain barrier, its brain concentrations are assumed to be proportional to plasma levels but somewhat lower due to metabolism and distribution [78]. Our results suggest that BSEP contains higher levels of SFN compared to other isothiocyanates, positioning it as a potentially rich source for SFN supplementation. Consequently, the plasma concentrations of SFN observed may be sufficient to induce the expression of protective enzymes, such as those regulated by the Nrf2 pathway, which is activated by SFN.

## 5 Conclusion

In this study, we optimized and patented (Korean) endogenous extraction protocol to maximize SFN yield in BSE. We introduced a non-organic solvent extraction method designed for industrial affordability, emphasizing endogenous SFN production to achieve stable SFN levels in BSE. Our findings demonstrate that BSEP exhibits good storage stability and consistency when refrigerated for over a month. Its high antioxidant capacity and its ability to alleviate inflammatory responses suggest potential health benefits of BSE containing high SFN as a supplement or therapeutic agent. Studies in AD-lesion induced mice further confirmed the neuroprotective effects of BSE, improving cognitive function and ameliorating impairment. SFN in BSE, known for its potent antioxidant properties, may protect neurons in Aβ-induced AD brains, despite no documented role in amyloidogenesis regulation. Thus, we propose an eco-friendlier form of SFN-rich BSE that could potentially meet daily SFN intake requirements, paving the way for SFN-based therapeutics at a commercial scale.

## CRediT authorship contribution statement

**Shyam Kokkattunivarthil Uthaman:** Writing – review & editing, Writing – original draft, Visualization, Validation, Software, Methodology, Investigation, Formal analysis, Data curation, Conceptualization. **Wan Seok Kang:** Visualization, Methodology, Investigation, Formal analysis, Data curation. **Ju-Young Park:** Investigation, Formal analysis, Data curation. **Sunoh Kim:** Writing – review & editing, Validation, Resources, Methodology. **Duc Dat Le:** Investigation, Data curation. **Suk-Jung Oh:** Writing – review & editing, Validation, Supervision, Resources, Methodology, Funding acquisition, Conceptualization. **Karthik Murugesh:** Writing – review & editing, Writing – original draft, Visualization, Software. **Laura Minju Oh:** Investigation, Formal analysis. **Mina Lee:** Writing – review & editing, Supervision, Resources. **Jin Woo Park:** Resources, Methodology, Funding acquisition.

## Ethics approval and consent to participate

This study was approved by the Experimental Animal Ethics Committees of B&Tech Co., Ltd. (BT-001-2023) and Mokpo National University (MNU-IACUC-2023-009).

## Availability of data statement

Data will be available upon request.

## Funding

This research was supported by 10.13039/501100003725National Research Foundation of Korea (NRF) grant funded by the 10.13039/501100014188Ministry of Science and ICT (MIST), Government of Korea (Grant No. RS-2022-NR070862).

## Declaration of competing interest

The authors declare that they have no known competing financial interests or personal relationships that could have appeared to influence the work reported in this paper.
