# Supplementary material for: Endogenous extraction yielded high quality sulforaphane from broccoli sprouts unveils potent antioxidant and anti-Alzheimer's activities
Source: Heliyon. 2025 Feb 13;11(4):e42673. doi: 10.1016/j.heliyon.2025.e42673 (PMC11875816; doi:10.1016/j.heliyon.2025.e42673)
Supplement: Multimedia component 1 [file mmc1.docx]

**Additional Information**

**Appendix I**

*Effect of BSEP solution on social interaction experiment*

Social interaction testing is a behavioral experimental technique that analyzes behavioral patterns similar to human social interaction in animal models. The experimental method involves placing an experimental animal and an unfamiliar animal (stranger) of the same type in the same space and analyzing the process by which the experimental animal encounters the unfamiliar animal and forms social interaction. Preference for social interaction or preference for new social interaction is determined by observing which box the experimental animal prefers in a situation where an unfamiliar animal is placed in one box and the other box is empty without an animal, using three interconnected boxes. When examining the effect of broccoli extract on social interaction, no aggressive behavior was observed, and the time spent with a new rat (stranger 2) increased compared to a familiar rat (stranger 1). As a result, it was confirmed that broccoli extract did not affect the social skills of mice.


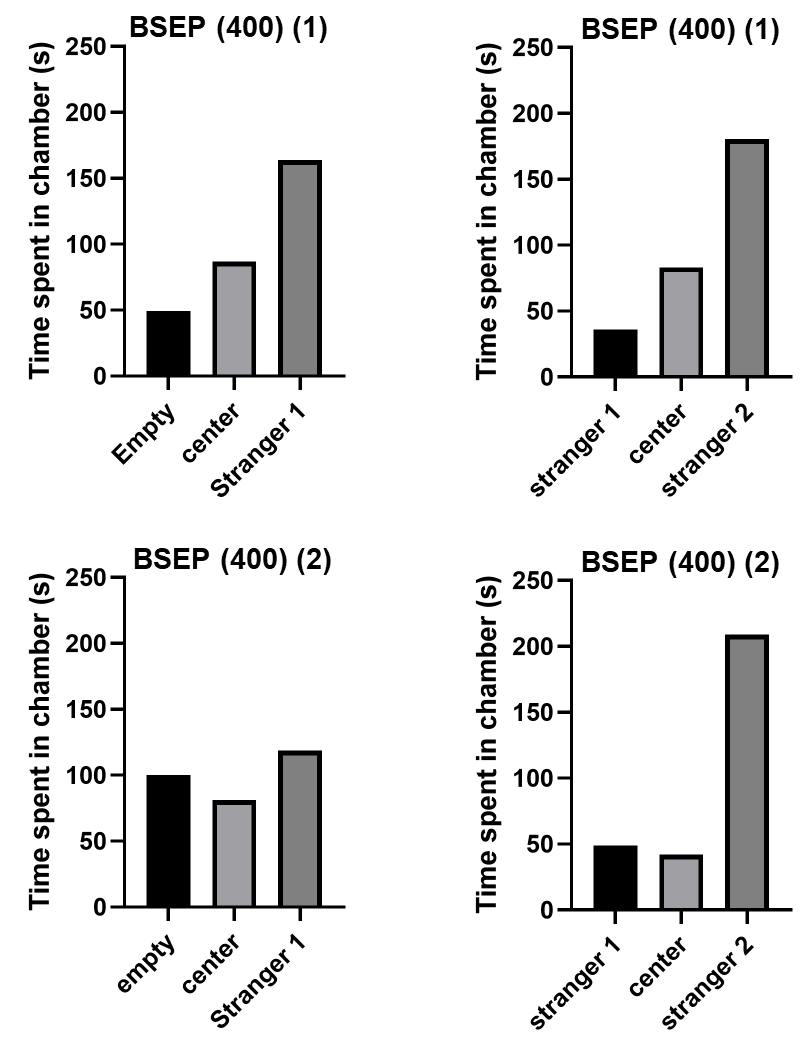


**Fig. S1.** Effect of BSEP solution on social interaction experiments in memory-impaired mice using amyloid beta (Aβ_1-42_).

**Appendix II**

*Retention test of Morris Water Maze*

In the Morris water maze test, after removing the platform on the final day, free swimming was conducted for 60 seconds, and this was recorded to measure the time spent in the quadrant where the platform was previously located out of the total time. The measurement results for each group were as follows: normal group: 10.72 ± 4.74 s, sham group: 13.81 ± 4.05 s, control group: 9.51 ± 5.2 s, experimental group 1: 13.59 ± 5.05 s, experimental group: 2 11.25 ± 4.57 s, positive control group 1: 16.26 ± 4.61 s, positive control group 2: 8.83 ± 5.92s. There was no clear significance compared to the control group. However, when comparing the control group with other experimental groups, there was a difference in the distance moved in the quadrant where the platform was located. Relative to the control group, the other experimental groups moved further in this quadrant. As the distance moved by mice in the other groups increased compared to the control group, it was inferred that they were actively searching for the platform. Therefore, there was no significant difference in the time spent in the quadrant.


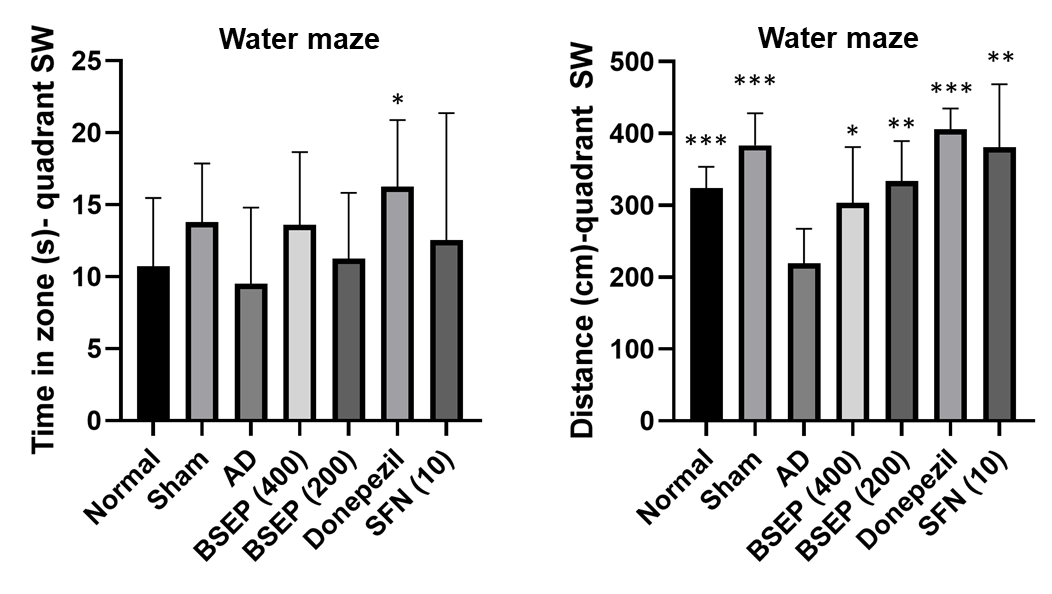


**Fig. S2.** In the Morris water maze experiment, the time a mouse with memory damage using amyloid beta (Aβ_1-42_) stayed in the quadrant where the platform was and the distance it moved. Data are presented as the mean ± SD (*n* = 7). ^*^p < 0.05, ^**^p < 0.01, ^***^p < 0.001 compared to the AD group.
